# Supplementary material for: Resveratrol Treatment Enhances the Cellular Response to Leptin by Increasing OBRb Content in Palmitate-Induced Steatotic HepG2 Cells
Source: Int J Mol Sci. 2019 Dec 12;20(24):6282. doi: 10.3390/ijms20246282 (PMC6941089; doi:10.3390/ijms20246282)
Supplement: Supplementary file 1 [file ijms-20-06282-s001.pdf]

Supplemental data for:

Resveratrol Treatment Enhances the Cellular Response to Leptin by Increasing OBRb Content in Palmitate-Induced Steatotic HepG2 Cells

Andrea Ardid-Ruiz [1], Maria Ibars [1], Pedro Mena [2], Daniele Del Rio [3,4,5], Begoña Muguerza [1], Lluís Arola [1,6] Gerard Aragonès [1,*] and Manuel Suárez [1]

[1] Universitat Rovira i Virgili, Department of Biochemistry and Biotechnology, Nutrigenomics Research Group, Tarragona, Spain; andrea.ardid@gmail.com (A.A.-R.); mibarsserra@gmail.com (M.I.); begona.muguerza@urv.cat (B.M.); lluis.arola@urv.cat (L.A.); gerard.aragones@urv.cat (G.A.); manuel.suarez@urv.cat (M.S.)

[2] Human Nutrition Unit, Department of Food and Drugs, University of Parma, Parma, Italy; pedromiguel.menaparreno@unipr.it (P.M.)

[3] Department of Veterinary Medicine, Human Nutrition Unit, University of Parma, Parma, Italy; daniele.delrio@unipr.it (D.DR.)

[4] School of Advanced Studies on Food and Nutrition, University of Parma, Parma, Italy

[5] Microbiome Research Hub, University of Parma, Parma, Italy

[6] Eurecat, Centre Tecnològic de Catalunya, Biotechnological Area, Reus, Spain

* Correspondence: gerard.aragones@urv.cat; Tel.: +34-977-558-188 (G.A.)

Table S1. Human-specific primer sequences used for qPCR analysis.

Name	Direction	Primer sequences (5′-3′)	Primer length (nucleotides)	Tm (ºC)
ACC	FW	gctgaaccagcactctgat	19	47
	RV	ccttgcacataaggtccagc	20	52
ATF4	FW	gggttctccagcgacaaggctaag	24	61
	RV	aacagggcatccaagtcgaactc	23	58
CHOP	FW	agggagaaccaggaaacggaaaca	24	61
	RV	tcctgcttgagccgttcattctct	24	60
CPT1a	FW	cgggggaatgtcaagaggtt	20	55
	RV	caagaaatgtcgcacgagcc	20	56
FAS	FW	tctactacaagctgcgtgcc	20	51
	RV	aacctctcccaggtatgcga	20	54
Il-6	FW	gcttccctcaggatgcttgt	20	51
	RV	attaactggggtgcctgctc	20	54
iNOS	FW	caagcagcagaatgagtccc	20	52
	RV	ctgggtcctctggtcaaact	20	50
OBRb	FW	aattgttcctgggcacaagg	20	54
	RV	gagagaccacagttgttggc	20	49
PPARα	FW	aggctgcaagggcttctttc	20	55
	RV	tcgggatgtcacacaacgc	19	55
PPIA	FW	ctcctttgagctgtttgcag	20	50
	RV	caccacatgcttgccatcc	19	54
PTP1b	FW	tgggaaatgcagggagttct	20	53
	RV	ccacgacccgacttctaact	20	51
SCD1	FW	acttggagctgtgggtgagg	20	54
	RV	cgctggcacatcaacttcac	20	54
SIRT1	FW	tggcacagatcctcgaacaa	20	54
	RV	catgaaacagacaccccagc	20	53
SOCS3	FW	caggaatgtagcagcgatggaa	22	57
	RV	cctgtccagcccaatacctga	21	56
sXBP1	FW	gagttaagacagcgcttggg	20	52
	RV	gatgttctggaggggtgaca	20	52
TNF-α	FW	gctgcactttggagtgatcg	20	52
	RV	gtgtgccagacaccctatct	20	53

Acc: acetyl-CoA carboxylase, *Atf4*: activating transcription factor 4, *Chop*: DNA damage inducible transcript 3, *Cpt1a*: carnitine palmitoyltransferase 1a, *Fas*: fatty acid synthase, *Il-6*: interleukine 6, *iNos*: inducible nitric oxide synthase, *ObRb*: leptin receptor isoform b, *Pparα*: peroxisome proliferator activated receptor α, *Ppia:* peptidylprolyl isomerase a, *Ptp1b*: protein-tyrosine phosphatase 1b, *Scd1*: stearoyl-CoA desaturase 1, *Sirt1*: NAD^{+}-dependent deacetylase sirtuin-1, *Socs3*: suppressor of cytokine signaling 3 and *sXbp1*: x-box binding protein 1, *Tnf-α*: tumor necrosis factor alpha.

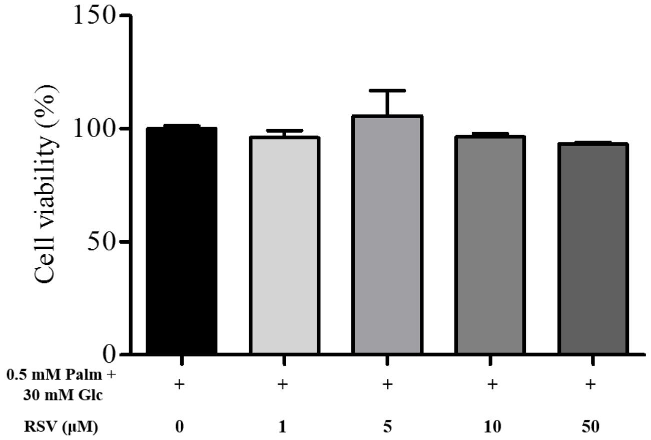

Figure S1. Cell viability by neutral red assay. Cells were incubated 48 h with Palm + Glc and the last 24 h were supplemented with 0, 1, 5, 10 and 50 μM RSV. Data are expressed as the mean ± SD of 3 replicates.

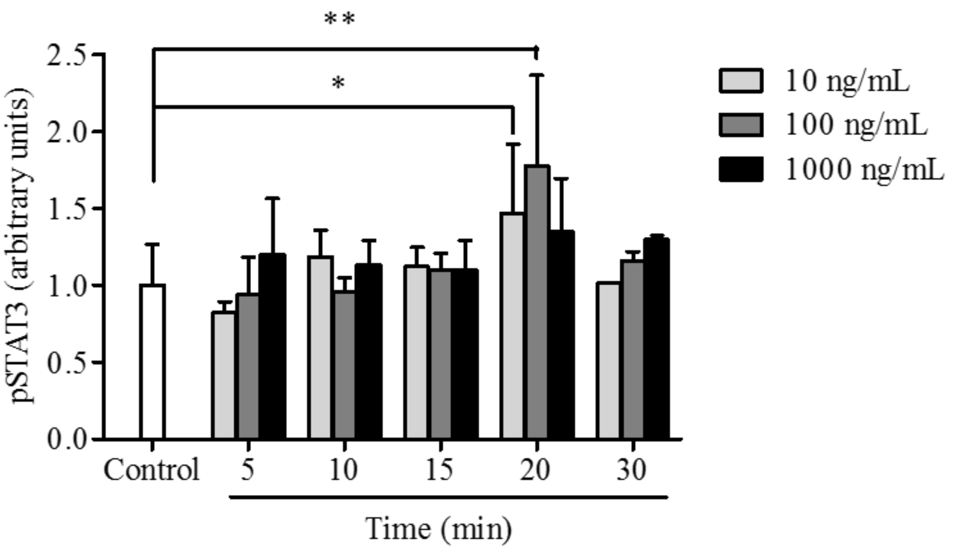

Figure S2. Activation of the leptin signalling pathway in HepG2 cells. pSTAT3 protein levels after the incubation of HepG2 cells with different leptin concentrations (10, 100 and 1,000 ng/mL) for different incubation times (5, 10, 15, 20 and 30 min). * $p < 0.05$ comparing leptin conditions to the control group as assessed by Student's t-test. Data are expressed as the mean ± SD of 3 replicates.

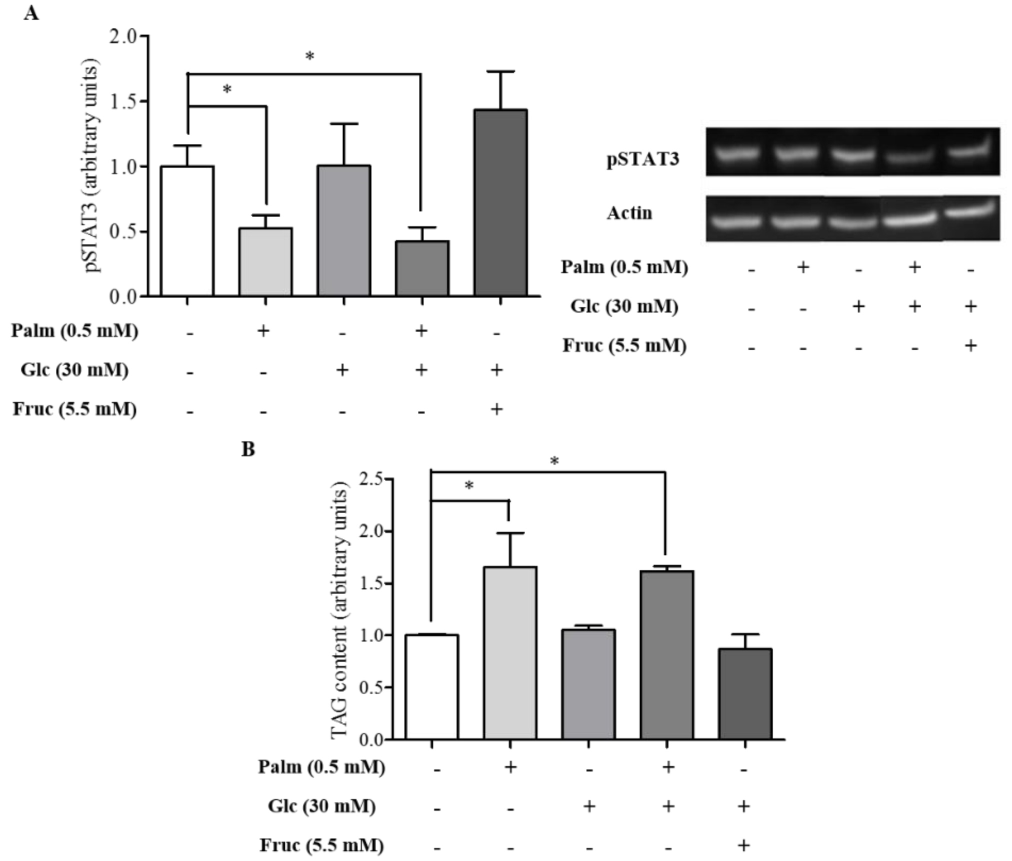

Figure S3. Development of a leptin resistance state in HepG2 cells**.** (**A**) pSTAT3 protein levels and (**B**) TAG content in HepG2 cells incubated with different treatments to produce leptin resistance. Cells were treated with 0.5 mM Palm, 30 mM Glc, 0.5 mM Palm + 30 mM Glc and 30 mM Glc + 5.5 mM Fruc for 48 h. Leptin signalling was activated by the addition of 10 ng/mL leptin during the last 20 min of incubation. $^* p < 0.05$ comparing treatment conditions to the control group as assessed by Student's t-test. Data are expressed as the mean ± SD of 3 replicates. A representative WB image for each RSV incubation time was included.

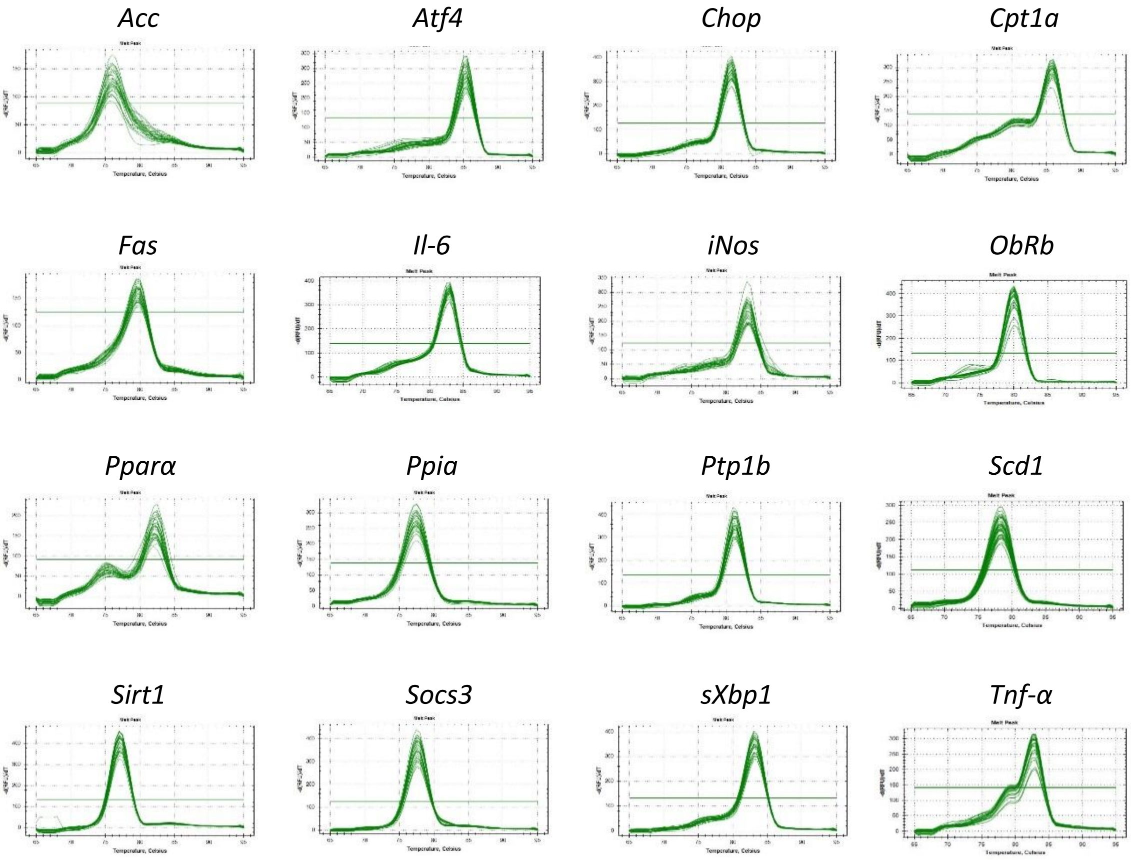

Figure S4. Melt curves from one RT-qPCR plate**.** The figure shows melt curves of primers in one plate for quantitative RT-PCR. Note that each primer has only one melt curve, indicating the specificity of the primers. *ACC*: acetyl-CoA carboxylase, *ATF4*: activating transcription factor 4, *CHOP*: DNA damage inducible transcript 3, *CPT1A*: carnitine palmitoyltransferase 1a, *FAS*: fatty acid synthase, *Il-6*: interleukine 6, *INOS*: inducible nitric oxide synthase, *OBRb*: leptin receptor isoform b, *PPARα*: peroxisome proliferator activated receptor *α*, *PPIA:* peptidylprolyl isomerase a, *PTB1B*: protein-tyrosine phosphatase 1b, *SCD1*: stearoyl-CoA desaturase 1, *SIRT1*: NAD^+-dependent deacetylase sirtuin-1, *SOCS3*: suppressor of cytokine signaling 3 and *SXBP1*: x-box binding protein 1, *TNF-α*: tumor necrosis factor alpha.
